# The pathogenesis and diagnosis of sepsis post burn injury

**DOI:** 10.1093/burnst/tkaa047

**Published:** 2021-02-04

**Authors:** Pengju Zhang, Bingwen Zou, Yih-Cherng Liou, Canhua Huang

**Affiliations:** 1 State Key Laboratory of Biotherapy and Cancer Center, West China Hospital, and West China School of Basic Medical Sciences & Forensic Medicine, Sichuan University, and Collaborative Innovation Center for Biotherapy, No.17 People’s South Road, Chengdu, 610041, China; 2 Department of Thoracic Oncology and Department of Radiation Oncology, Cancer Center, West China Hospital, Sichuan University, No.37 Guoxue Alley, Wuhou District, Chengdu, 610041, China; 3 Department of Biological Sciences, Faculty of Science, National University of Singapore, 14 Science Drive 4, 117543, Singapore

**Keywords:** Burn, Infection, Sepsis, Septic shock, Multiple organ dysfunction syndrome, Immune dysregulation, Hypermetabolism, Trauma, Biomaker, Inflammation

## Abstract

Burn is an under-appreciated trauma that is associated with unacceptably high morbidity and mortality. Although the survival rate after devastating burn injuries has continued to increase in previous decades due to medical advances in burn wound care, nutritional and fluid resuscitation and improved infection control practices, there are still large numbers of patients at a high risk of death. One of the most common complications of burn is sepsis, which is defined as “severe organ dysfunction attributed to host's disordered response to infection” and is the primary cause of death in burn patients. Indeed, burn injuries are accompanied by a series of events that lead to sepsis and multiple organ dysfunction syndrome, such as a hypovolaemic state, immune and inflammatory responses and metabolic changes. Therefore, clear diagnostic criteria and predictive biomarkers are especially important in the prevention and treatment of sepsis and septic shock. In this review, we focus on the pathogenesis of burn wound infection and the post-burn events leading to sepsis. Moreover, the clinical and promising biomarkers of burn sepsis will also be summarized.

Highlights

- Sepsis is one of the most common and severe complications of severe burns.
- Immunosuppression and hypermetabolism play key roles in the development of burn sepsis.
- Recent diagnostic tools and potential biomarkers are discussed.

## Background

**Figure 1.**
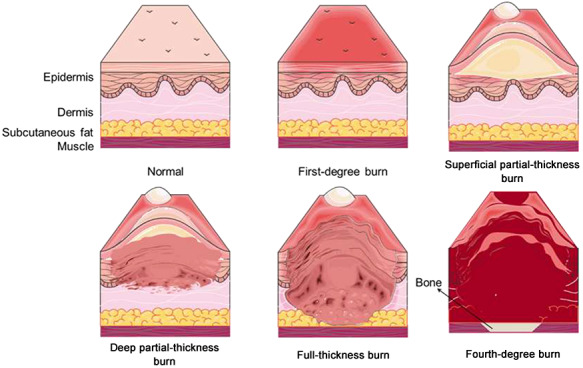
Zones of burn injury for different depths. First-degree burns only involve the epidermis (the uppermost layer of the skin); the skin becomes red and painful, but this is limited in duration. Burns that affect the dermis (the underlying skin layer) are classed as partial-thickness burns, which are frequently accompanied by the formation of painful blisters that increase the risk of infection. Partial-thickness burns can be divided into superficial partial-thickness burns, which are painful, moist, hyperemic and blanch, and deep partial-thickness burns which are less sensate, drier and do not blanch. Full-thickness burns extend through the full dermis and require surgical management due to high risk of infection. Burns extending into deeper tissues (such as muscle or even bone) are defined as fourth-degree burns and are usually blackened and often result in loss of the burned tissues

**Figure 2.**
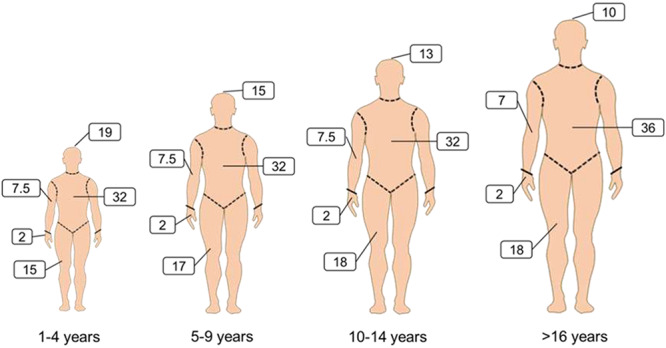
Lund and Browder diagrams for estimation of total burned surface area (TBSA). The “rule of nines” (using multiples of 9) is frequently used to assess the proportion of TBSA affected in adults and to help guide immediate treatment decisions based on burn size. However, the rule of nines is inaccurate in children due to different head-to-body size ratios at different ages. Lund and Browder diagrams are therefore more suitable for assessing of the proportion of TBSA affected in both children and adults. The body areas are separated into different regions (including anterior and posterior) by dashed lines and the numbers are percentages of the TBSA. For instance, 19 in the diagram for children aged 1–4 years relates to the face, neck and head that make up 19% of theTBSA

Burn injuries cause unpredictable and devastating trauma and are associated with high morbidity and mortality. There are numerous causative mechanisms, including physical (friction, high temperature, cold, radiation and electricity) and chemical factors [1]. Nevertheless, thermal injury caused by hot liquids, solids or fire makes up the majority of burn injuries [2]. According to a report from the World Health Organization in 2018, about 11 million burn cases occur annually worldwide, with burn injuries claiming as many as 180,000 lives [3]; looking back to almost a decade ago, mortality from burns has decreased from the 300,000 deaths recorded in 2011 [4]. The significant improvement in the survival rate of burn patients is in part attributed to advances in intensive care unit treatment and improved wound management, infection control practices and control of hemodynamic disorders [5, 6]. The mortality rate, however, remains unacceptably high, particularly in patients with severe burns. The severity and prognosis of burn injuries depends principally on the depth (Figure 1) and size (Figure 2) of the burn site. Most patients who suffer from severe burn injuries require rapid and specialized emergency burn care to reduce morbidity and mortality. The high fatality rate of severe burns is due to not only hypovolaemic shock and vascular leak, but also abnormal body responses, including immunosuppression [6, 7], excessive inflammation [8] and hypermetabolism [9]. These responses that accompany severe burn injury will result in increased incidence of infection, sepsis and multiple organ dysfunction syndrome (MODS), which are the leading causes of death in severe burn patients [10].

Burn wound infection is one of the most common and severe complications of severe burns, and occurs due to profound hypermetabolic response and the loss of skin, which is considered the first line of defense against microbial invasion in hosts [11]. Under the conditions of a dysregulated host response to an infection, burn patients may develop sepsis syndrome characterized by fever, increased fluid requirements, decreased urinary output and even MODS [12–14]. In 2016, the Third International Consensus Definition for Sepsis and Septic Shock (Sepsis-3) redefined sepsis as life-threatening organ dysfunction caused by a dysregulated host response to infection [15]. This new sepsis definition places more emphasis on the process of organ dysfunction, and the Sequential Organ Failure Assessment score (Table 1) is used to define sepsis severity, including septic shock [15, 16]. Indeed, the incidence of sepsis in burn patients can range between 3–30% for burns of more than 20% of the total body surface area (TBSA) [17]. Even more concerning is that approximately 54% of burn-related deaths in modern burn units occur due to septic shock and MODS instead of osmotic shock and hypovolemia [18, 19]. A recent autopsy study showed that over 60% of deaths in burn patients resulted from infectious complications and MODS, which is a direct consequence and poor outcome of sepsis [20]. Therefore, the early diagnosis and effective treatment of sepsis would benefit burn patients, especially those with severe burns. Although the Surviving Sepsis Campaign has put in immense effort to drive the improvement of survival in sepsis and septic shock patients, burn wound sepsis is distinguished from general sepsis because of skin loss that suggests the risk of infection is present as long as the burn wounds have not healed [21, 23].

**Table 1:** Sequential organ failure assessment (SOFA) scoring system [37]

**Six organ systems**	**SOFA score 0**	**SOFA score 1**	**SOFA score 2**	**SOFA score 3**	**SOFA score 4**
Respiratory system: PaO_2_/FiO_2_ (kPa)	≥53.3	<53.3	<40	<26.7	<13.3
Coagulation system: platelets (× 10^3^/μL)	≥150	<150	<100	<50	<20
Hepatic system: bilirubin (μmol/L)	<20	20–32	33–101	102–204	>204
Cardiovascular system^a^	MAP>70 mm Hg	MAP<70 mm Hg	Dopamine ≤5 or dobutamine (any dose)	Dopamine >5 or epinephrine ≤0.1 or norepinephrine ≤0.1	Dopamine >15 or epinephrine >0.1 or norepinephrine >0.1
Central nervous system: Glasgow Coma Scale	15	13–14	10–12	6–9	<6
Renal system Creatinine (μmol/l) Urine output (ml/day)	<100	111–170	171–299	300–440< 500	>440< 200

a Adrenergic agents administered for at least 1 h (doses given are in μg/kg^**.**^ min) *PaO_2_* partial pressure of arterial oxygen, *FiO_2_* fraction of inspired oxygen, *MAP* mean arterial pressure

In this review, we seek to address the major pathogenesis of current categories of infection, sepsis and septic shock in patients with burn injury. In addition, the recent diagnostic tools and potential biomarkers, including C-reactive protein (CRP), procalcitonin (PCT) and cytokines, are intensively discussed.

## Review

### Burn wound infections

Burn wound infection, one of the most important causes of sepsis, is associated with high fatality rates in patients with burn injury. The occurrence of burn wound infection often surfaces during the acute post-injury period and exhibits considerable differences among burn patients of different ages [21, 24]. Young children (under the age of 4 years) and elderly adults (over the age of 55 years) have a higher risk of being infected, with higher fatality rates compared with other age groups [25, 26]. A possible cause is that infants, young children and the elderly have an increased inclination for deep burn injury due to their much thinner dermal layer [27]. Another reason may be the poor compliance of these patients with early medical care and drug regimens. Apart from the above, some special populations, including obese adults, diabetes patients and AIDS patients, have a higher incidence of burn wound infection and have also been shown to have more complications related to infection [28, 29]. For example, AIDS patients have a higher incidence of sepsis and a longer period of hospitalization than HIV-negative patients, although data on the reported outcome are limited due to the small number of AIDS patients [30].

Indeed, burn wound infection can be considered a series of dynamic pathophysiological processes, including microbial colonization, biofilm formation and invasive burn wound infection. Microorganisms can rapidly colonize the burn wounds due to thermal destruction of the skin barrier. Burn eschar (avascular necrotic tissue) caused by deep partial-thickness and full-thickness burns provides a protein-rich niche for bacterial colonization and proliferation [31, 32]. In addition, burn eschar may also increase the risk of infection by inhibiting early healing via basic fibroblast growth factor-induced endothelial cell proliferation and sprouting [33]. However, eschar factors can inhibit hypertrophic scar formation of full-thickness burn wounds by preventing excessive granulation tissue formation [33].

Once planktonic (free-living) organisms form aggregates and attach to burn wounds, the formation of biofilm is initiated. Biofilms are defined as structured communities encased in a self-produced extracellular polysaccharide matrix, or slime [34, 35]. A mature biofilm provides efficient barriers for microorganisms against the host immune system and antimicrobial agents, including biocides, antibiotics, oxidizing agents and nano-drugs [36]. For example, the microorganisms within biofilms have an increased capacity to tolerate and survive stressful environments (such as nutrient deprivation, hypoxia, dehydration and pH changes) compared to planktonic microorganisms [38]. All these resistances pose huge challenges for eliminating antibiotic-resistant microbes and preventing burn wound sepsis. In fact, biofilm formation is a sequential cyclic process comprising at least 5 phenotypically distinct stages, including attachment of planktonic cells, aggregation, biofilm maturation, establishment of cells with the biofilm subpopulation and biofilm dispersion. The biofilm dispersion refers to the process by which cells with biofilm subpopulation escape from the biofilm structure [38]. Although biofilm dispersal leads to the loss of multiple survival advantages, dispersion facilitates the formation of channels on the biofilm surface. These channels contribute to the removal of metabolic waste and the intake of nutrient resources and oxygen [39, 40]. Notably, Kennedy and colleagues detected biofilms not only in ulcerated areas of the burn wound, but also in bacterial wounds invaded with mixed organisms [41]. Therefore, the formation of biofilms may promote the development of an invasive wound infection and, to some extent, sepsis, although the mechanisms remain unclear.

The risk of invasive burn wound infection depends on the surface area and depth of the burn wounds, host immunity and the types (virulence difference) and amount of microbial flora colonizing the burn wounds. Microorganisms causing burn wound infection include gram-positive bacteria, gram-negative bacteria, fungi and viruses (Table 2). During the initial stage of burn injury, some gram-positive bacteria (such as the *Staphylococci spp.* derived from endogenous skin, gastrointestinal and respiratory flora, or the external environments) are vanguard microbes colonizing the burn wounds [31, 42]. Globally, the major cause of early burn wound infection is by *Staphylococcus aureus (S. aureus)*, which also plays an important role in invasive burn wound infection and sepsis [43]. There are various genes that encode molecules associated with virulence factors, including cell-surface virulence factors (capsular polysaccharides, cell wall anchored proteins and lipoteichoic acids) [44, 45] and secreted virulence factors (superantigens [46], cytotoxins, exoenzymes and miscellaneous proteins [47]) in the *S. aureus* genome, including the core genome and accessory genome. Although knockouts of these genes have no identifiable effect on the growth of organisms in vitro, their pathogenicity may be diminished in vivo [48, 49]. In addition, these virulence factors can facilitate the adherence of organisms to host tissues, invasion of host cells and tissues and evasion of the host immune system [50]. For example, clumping factor A, a member of the microbial surface components recognizing adhesive matrix molecules family, has been demonstrated to play key roles in sepsis in the murine model [51, 52].

**Table 2:** Common microorganisms causing invasive burn wound infection

**Group**	**Species**	**References**
Gram-positive organisms	*Staphylococcus aureus* Methicillin-resistant *Staphylococcus aureus* Coagulase-negative Staphylococci	[27] [161] [162]
Gram-negative organisms	*Pseudomonas aeruginosa* *Escherichia coli* *Klebsiella pneumoniae*	[53] [163] [164]
Fungi	*Candida* spp. *Aspergillus* spp. *Mucor* spp.	[43] [165] [166]
Viruses	Herpes simplex virus Cytomegalovirus Varicella-zoster virus	[167] [168] [169]

In the first 5–7 days after injury, burn wounds are occupied by other microorganisms, such as gram-negative bacteria, fungi and viruses. For example, *Pseudomonas aeruginosa* (*P. aeruginosa*), a gram-negative organism, is a common culprit of burn wound infection in the intensive care unit due to their multi drug resistances and multiple virulence factors [53, 54]. In a study of *P. aeruginosa* prevalence in Chinese burn wards from 2007 to 2014, Dou and coworkers showed that the detection rate of *P. aeruginosa* in hospitalized burn patients increased from 10.20% in 2007 to 26.16% in 2014 [55]. The main cause of this growing trend may be the metabolic versatility of *P. aeruginosa*, its ability to colonize of a wide range of ecological niches and its low outer membrane permeability, which can resist antiseptics and antibiotics [56]. Similar to *S. aureus*, *P. aeruginosa* also has quite a lot of virulence factors, including adhesins, lipopolysaccharides, elastases, exoenzyme S, exotoxin A, leukocidins and proteases. These make *P. aeruginosa* a major cause of bloodstream invasion, sepsis and poor prognosis in severely burned patients [57, 58]. It remains unclear whether the formation of biofilm or invasive burn wound infection is the important inducer for sepsis. Therefore, the prevention of burn wound infection remains the better choice to diminish the incidence of sepsis and septic shock.

### Events leading to sepsis and septic shock following burn injury

**Figure 3.**
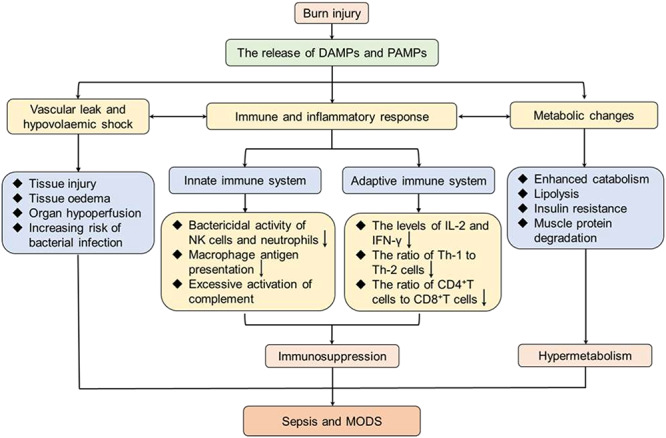
A series of pathogenic events responsible for sepsis post burn injury. Following severe burn injury, damaged tissues lead to the release of endogenous DAMPs (such as double-stranded RNA and mitochondrial DNA) and exogenous PAMPs (such as lipopolysaccharides and peptidoglycans). Subsequently, PAMPs can result in vascular leak and hypovolemic shock, immune and inflammatory responses and metabolic changes. Vascular leak causes tissue edema, organ hypoperfusion and increasing risk of bacterial infection. Meanwhile, the excessive inflammatory response leads to immunosuppression by inhibition of the innate and adaptive immune systems. Moreover, hypermetabolism emerges in the form of enhanced catabolism, lipolysis, insulin resistance and muscle protein degradation. These events contribute to the susceptibility of the burn patients to sepsis and MODS. *DAMPs* damage-associated molecular patterns, *PAMPs* pathogen-associated molecular pattern molecules, *NK* natural killer, *IL-2* interleukin 2, *IFN-γ* interferon γ, *Th-1* helper T lymphocyte 1, *Th-2* helper T lymphocyte 2, *MODS* multiple organ dysfunction syndrome

**Figure 4.**
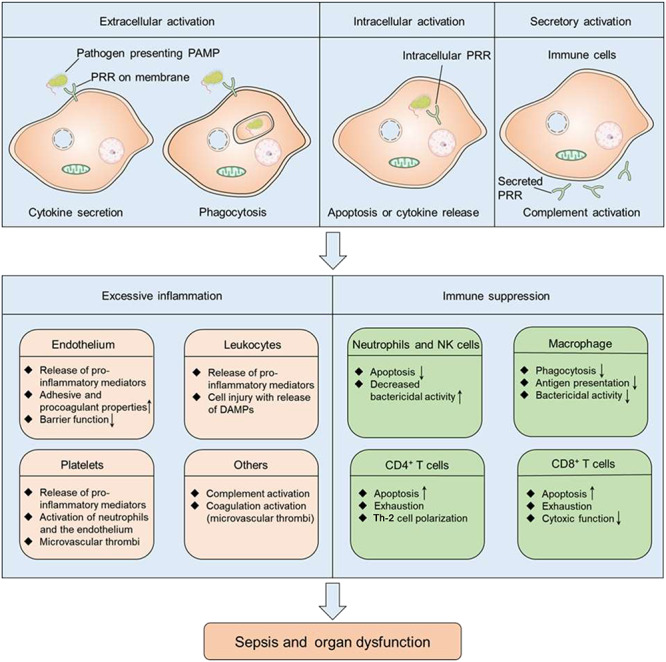
The host response to infection and during sepsis. After infection, PAMPs are released and interact with cell-surface, intracellular and even secreted PRRs, including toll-like receptors, nucleotide-binding oligomerization domain-like receptors, retinoic acid-inducible gene-like receptors and C-type lectin receptors. The interaction between PAMPs and PRRs can result in cytokine secretion, immune cell apoptosis and the activation of the complement system. In some burn patients, these events lead to the simultaneous imbalanced activation of proinflammatory response (excessive inflammation) and anti-inflammatory response (immune suppression). Excessive inflammation can result in the dysfunction of the endothelial barrier, microvascular thrombi and further injuries. Immunosuppression causes decreased bactericidal activity of neutrophils and NK cells, decreased phagocytosis and antigen presentation of macrophages and impaired innate immune system response. Taken together, excessive inflammation and immunosuppression contribute to a greatly increased risk for sepsis and organ dysfunction. *PAMPs* pathogen-associated molecular pattern molecules, *PRR* pattern recognition receptors, *DAMPs* damage-associated molecular patterns, *NK* natural killer, *Th-2* helper T lymphocyte 2

Sepsis and MODS are common complications of invasive burn infection and are responsible for a significant proportion of the mortality in patients with burn injuries, particularly severe burns. Williams *et al.* performed statistical analysis on patients with burn injury and found that 55% of males and 54% of females died from sepsis and infections between 1989 to 2009, but the data short of highlighting the global prevalence of this trend [59]. The limitation is attributed mainly by neglecting low-income and middle-income countries, which may have a higher incidence of sepsis in burn patients. Therefore, clear diagnostic criteria for burn sepsis are necessary to minimize and prevent septic complications. The updated criterion in the Sepsis-3 consensus definition established in 2016 focuses more on multiple organ dysfunction than on signs of inflammation [15], compared with the American Burn Association (ABA) sepsis criteria (2007) [60] and the Mann-Salinas novel burn-specific sepsis predictors (2013) [61, 62] (Table 3). This is particularly discerning, considering that a series of pathophysiological events can lead to sepsis and multiple organ failure, including inflammatory response, hypovolaemic shock and vascular leak, immune dysregulation and hypermetabolism (Figure 3), with inflammation present almost throughout the whole process from initial injury to burn wound healing. Inflammation behaves like a double-edged sword in burn injuries: immediately following minor burn injuries, inflammatory responses are initiated to activate the cascade of signals required for wound healing [8]; however, in patients with severe burns, the inflammatory response (Figure 4) is uncontrolled and leads to vascular endothelium dysfunction, delayed healing, immune suppression and systemic inflammatory response syndrome [63]. Metabolically, inflammation also causes an enhanced catabolic state that is associated with an increased incidence of sepsis and multiple organ failure. Compared with patients with only burns, the level of catabolism in septic burn patients is more than doubled, as measured using stable isotope perfusion [64].

**Table 3:** Different criteria for sepsis

**Consensus definitions**	**Criteria**	**Predictors**
ABA Sepsis Criteria [59]	At least one or more of the following	1) Positive culture 2) Pathologic tissue source identified 3) Clinical response to antimicrobial agents
	AND at least three of the following predictors	1) Temperature >39 °C or <36.5 °C 2) Progressive tachycardia (>110 bpm) 3) Progressive tachypnoea 4) Thrombocytopenia 5) Hyperglycaemia 6) Inability to continue enteral feedings 24 hours
Mann-Salinas et al. Novel burn-specific sepsis predictors [60, 61]	Predictors	1) Tachycardia >130 bpm 2) MAP <60 mmHg 3) Base deficit <–6 mEq/l 4) Hypothermia <36 °C 5) Use of vasoactive medications 6) Hyperglycaemia >150 mg/dl
Sepsis-3 Consensus definition for sepsis^a^ [15]	qSOFA score ≥ 2	1) Altered mental status (Glasgow Coma Scale <13) 2) Systolic blood pressure ≤100mmHg 3) Respiratory rate 22 ≥ breaths/min
	SOFA variables ≥ 2	1) PaO_2_/FiO_2_ ratio 2) Platelet count 3) Bilirubin 4) Mean arterial pressure 5) Glasgow Coma Scale 6) Vasopressor requirement 7) Serum creatinine or urine output
	Septic shock predictors (sepsis and both predictors)	1) Vasopressors required to maintain MAP >65mm Hg 2) Lactate >2 mmol/L (after adequate fluid resuscitation)

^a^Suspected or documented infection and qSOFA ≥ 2 and/or SOFA ≥ 2 *ABA* American Burn Association, *MAP* mean arterial pressure, *bpm* beats per minute, *SOFA* Sequential Organ Failure Assessment, *qSOFA* quick SOFA, *PaO_2_* partial pressure of arterial oxygen, *FiO_2_* fraction of inspired oxygen

### Vascular leak and hypovolemic state

Interconnected microvessels are crucial for substance exchange (nutrients, oxygen and metabolic waste) between blood and surrounding tissues through the regulation of local hydrostatic and oncotic pressures [65]. Under the regulation of physical structure and chemical messenger, the vascular barrier function maintains tissue perfusion and homeostasis to adapt to physiological stimuli. The vascular barrier function maintains tissue perfusion and homeostasis to adapt to physiological stimuli under the regulation of physical structure and chemical messenger. However, in some traumatic injuries, particularly burn injuries, structural disruption and inflammatory mediators lead to increased vascular permeability, which contributes to leakage of the intravascular fluids into the interstitial space, leading to further profound tissue edema and even hypovolemic shock [66].

Multiple mediators of barrier function, including histamine, bradykinin, platelet-activating factor, leukotrienes, vascular endothelial growth factor (VEGF) and VEGF receptors, affect vascular hyperpermeability by inducing cellular signaling and structural alterations. Histamine is released mainly from mast cells and increases vascular permeability through vascular dilation, increasing of blood flow and endothelial barrier disruption. There is increasing evidence that nitric oxide (NO) and RhoA/Rho-associated protein kinase (ROCK) play an important role in histamine-induced hyperpermeability. Ashina and coworkers found that inhibiting NO synthesis with a nitric oxide synthase (NOS) inhibitor, Nitro-L-arginine methyl ester (L-NAME) could alleviate the histamine-induced blood flow increase and hyperpermeability [67]. In addition, the authors also found that histamine disrupted the endothelial barrier due to the localization of vascular endothelial cadherin (VE-cadherin) (a cadherin maintaining the junction of adjacent endothelial cells) at the endothelial cell junction being changed [67]. Consistent with this observation, Mikelis *et al.* suggested that histamine induced the localization of VE-cadherin adhesion complexes to focal adherens junctions through Rho/ROCK and further led to the formation of gaps in the endothelial barrier [68].

Bradykinin, like histamine, activates RhoA and induces the rearrangement of the cytoskeleton and disassembly of tight junction (TJ) proteins. These changes can cause vascular leakage, although vascular hyperpermeability was observed in the blood–tumor barrier of rat brain microvascular endothelial cells [69]. Furthermore, previous studies have shown that bradykinin downregulates the expression of the TJ-associated proteins zonula occluden-1, occludin and caludin-5, but the regulatory mechanism might involve the cyclic adenosine monophosphate (cAMP)/protein kinase A (PKA) signal transduction system rather than the RhoA/ROCK-dependent mechanism [70].

Surprisingly, cysteinyl leukotrienes also directly regulate vascular permeability through the activation of ROCK to promote endothelial contraction and gap formation [71]. Taken together, these studies suggest that RhoA signaling circuitry plays a key role in vascular permeability and RhoA/ROCK is a potential pharmacological target for burn-induced vascular leakage. Indeed, in most cases, leukotrienes act as an upstream regulator to influence vascular permeability by regulating other vasoactive mediators, typically VEGF [72]. VEGF signaling regulates vascular permeability by a variety of mechanisms, including reductions in TJ-associated proteins [73], damage of VE-cadherin junctional contacts [74] and the formation of vesicular vacuolar organelles [75]. Vesicular vacuolar organelles allow endothelial cells to form transendothelial cell pores, which are considered an additional transcellular pathway for large molecules and fluid extravasation [76]. VEGF has been used as a biomarker for the diagnosis of sepsis in patients with severe burn injuries [77].

Apart from vascular leakage, burn injuries also result in cardiac dysfunction due to cardiac mitochondrial damage [78] and the production of inflammatory mediators, including macrophage migration inhibitor factor [79] and tumor necrosis factor (TNF) [80]. In rat models of burn, oxidative stress impairs cardiac function due to a 30–50% increase in lipid peroxidation in cardiac mitochondria [78]. Migration inhibitor factor is released by the skin and cardiomyocytes and is considered a critical mediator of persistent cardiac dysfunction [79]. The cardiac dysfunction and vascular leakage-induced hypovolemia have serious implications for the perfusion of tissues and organs (lungs, liver, kidney and gastrointestinal tract) and may even lead to sepsis and multiple organ failure.

### Immune dysregulation

As discussed above, patients with burns have a greater risk of infection, not exclusively due to the loss of the natural barriers function of the skin. Recent studies have demonstrated that burn injuries can also influence other elements of both the innate immune system (including immune cells and complement) and the adaptive immune system, which disrupts coordination of the immune response. Some immune cells, including monocytes, macrophages, dendritic cells, natural killer (NK) cells and neutrophils, are among the first immune cells to respond to wounds and coordinate the wider immune response. Following burn injuries, the antimicrobial actions of neutrophils and NK cells are impaired [81–83]. Coincidentally, the phagocytic capacity of macrophages is also diminished in severe burn [84]. In addition, increased apoptosis of conventional and plasmacytoid dendritic cells has been observed in patients with sepsis [85]. The complement system represents an evolutionarily conserved and important element of the innate immune system [85]. According to the severity of burns, the levels of complement decrease to different extents at the beginning of burn injury and subsequently rise to unprecedented levels [87]. The increased complements, such as C3a, C3b and C5a, may suppress immune response directly by impairing the function of leukocytes and lymphocytes [88, 89]. Intriguingly, multiple interleukins (IL, a kind of lymphokine), such as IL-4 and IL-10, can significantly inhibit the antigen presentation of macrophage and the bactericidal activity of NK cells and neutrophils [90–92].

In addition to impairing the function of the innate immune system, severe burns reduce the total numbers of T lymphocytes, which play dominant roles in the adaptive immune system [93, 94]. Surprisingly, not all T lymphocytes are diminished, with helper T lymphocyte 2 (Th-2) present in increased numbers due to the increased levels of IL-4 and IL-10 [95]. Moreover, the reduced levels of IL-2 and interferon-γ also cause the increase of Th-2 following burn injuries [96]. The depressed levels of IL-2 and interferon-γ and high levels of IL-4 and IL-10 simultaneously inhibit the activity of helper T lymphocyte (Th-1) that support cell-mediated immune responses [95]. The decreased ratio of Th-1 to Th-2 is an important etiologic factor in the suppression of adaptive immune responses [27]. Furthermore, the ratio of CD4-positive T helper cells to CD8-positive T suppressor cells also declines after severe burn [97]. Similarly, burn injury results in immune dysregulation by destabilizing the balance between helper T lymphocyte 17 (Th-17) and regulatory T cell, which plays prominent roles in protection against bacterial infections. On the one hand, Th-17 responses have been shown to be elicited in murine models of burn injury [98]. The perturbation of Th-17 cytokines IL-17 and IL-22 may further delay wound healing and promote burn sepsis [99]. On the other hand, the proportion of Treg cells is increased in patients with burn injury and this may decrease effector T cell function and further contribute to sepsis [100]. Taken together, the compromised alterations in innate and adaptive immune responses result in enhanced susceptibility to infection, sepsis and multiple organ failure.

### Hypermetabolic state in bury injury

In addition to hypovolemic response and immune dysfunction, the hypermetabolic state following burn trauma is another primary contributor to multiple organ failure and sepsis. Hypermetabolism (increased metabolic rate) is characterized by an elevated (>10% above normal) resting energy expenditure [101] and is more likely to occur in severe burns (>20% TBSA). Studies have demonstrated that there is an increase of 40–80% in resting energy expenditure during the acute phase of post-burn injury in patients with burns of more than 40% TBSA [102, 103]. Generally, the metabolic level is attenuated in the early stage of burn (<48 hours) owing to diminished cardiac output and oxygen consumption, but metabolism will enhance dramatically after the “ebb” phase [104]. Transient hypermetabolic state has proven to be beneficial to burn patients. For example, hypermetabolism can provide more energy for vital organs (brain, lungs, heart and immune organs) to maintain their functional levels as closely as possible to normal physiological conditions. Nevertheless, as distinguished from other trauma, major burns provoke profound hypermetabolism and the resultant hypermetabolic state can persist for up to and beyond 36 months after the initial insult [9]. Persistent hypermetabolism aggrandizes the rates of glycolysis, lipolysis and proteolysis, and subsequently results in muscle wasting and loss of body weight that can significantly impact the immune response and wound healing [8]. The mechanisms underlying burn-induced hypermetabolism are associated with many factors, including pro-inflammatory cytokines, stress hormones, mitochondrial dysfunction, endoplasmic reticulum stress and the browning of white adipose tissue (WAT).

Pro-inflammatory cytokines not only contribute to immune dysfunction in the burn patients but also induce hypermetabolism. Levels of cytokines, including TNF-α and IL-1β, do not increase sustainedly, but are restricted to the acute phase of burn [9, 63, 105]. The mechanism of TNF-α-induced hypermetabolism may be that TNF-α promotes the production of reactive oxygen species, adipose catabolism and the release of free fatty acids in patients suffering from thermal injuries [106]. In addition to TNF-α, recent studies have postulated that IL-1β contributes to the hypermetabolic state following burn injuries because it interferes with insulin sensitivity by inhibiting the expression of insulin receptor substrate-1 interfere with insulin sensitivity [104, 107]. Compared with pro-inflammatory cytokines, stress hormones (such as epinephrine, norepinephrine, glucagon and cortisol) have profound and enduring impacts on hypermetabolism. Incremental levels of these stress hormones have pleiotropic hypermetabolic effects that enhance lipolysis, proteolysis and glucose metabolism by acting on several target organs, such as adipose tissue, skeletal muscle and the liver [63, 108–110]. Moreover, accumulating evidence indicates that the functional changes of some organelles, such as mitochondria, can contribute to the hypermetabolic response to burns. Several adenosine triphosphate (ATP)-consuming reactions are enhanced in response to burn injury, including protein synthesis, hepatic gluconeogenesis and cycling of glucose and fatty acids [111]. About 57% of the increase of energy expenditure in severely burned patients is attributed to these ATP consuming reactions [111]. Mitochondria, as the powerhouse of cells, play an important role in ATP production, mainly via the coupling of oxidative phosphorylation. However, the coupling of mitochondrial respiration to adenosine diphosphate phosphorylation is significantly attenuated in patients with burns [101]. On the contrary, the uncoupling of oxidative phosphorylation, which contributes to proton conductance via uncoupling proteins for mitochondrial thermogenesis rather than generation of ATP, is enhanced post burn [112]. Hypermetabolism induced by burn injuries imposes an immense burden on the endoplasmic reticulum (ER) and leads to the accumulation of unfolded proteins, which are responsible for ER stress through protein synthesis demand and extracellular signaling [104, 113]. Interestingly, ER stress in turn inhibits WAT browning, which is the emerging culprit of hypermetabolic response, although the mechanism remains unclear [114, 115]. The browning of WAT refers to the conversion of WAT into brown adipose tissue, characterized by higher rates of lipolysis. Following a thermal injury, the browning of WAT is enhanced and increases the circulation of free fatty acids implicated in the hypermetabolic state [116]. The browning of WAT can also be induced by IL-6 [117] and uncoupling protein 1 of mitochondria [118]. Taken together, the browning of WAT is a valuable therapeutic target and needs more mechanistic studies associated with browning and burn-induced hypermetabolism to further understand its function.

### Septic shock in burn injury

In cases of burn wound infection, a hypovolemic state, dysregulated inflammatory response and hypermetabolism are the primary risk factors for the development of sepsis. If not diagnosed in time and treated appropriately, burn patients with sepsis may progress to severe sepsis or septic shock, which is defined as sepsis with intravascular hypovolemia and hypotension resistant to fluid resuscitation, accompanied by worsening systemic signs, including oliguria, lactic acidosis and even changes in mental status [119, 120]. Under normal physiological conditions, there is a delicate balance between procoagulant and anticoagulant mediators within the vasculature. In severe sepsis, however, several proinflammatory cytokines (including IL-1, IL-6 and TNF-α), induced by endotoxins, lipopolysaccharides and other infectious mediators, promote the generation and release of procoagulant tissue factor of endothelial cells [121]. The increase of procoagulant tissue factor disrupts vascular homeostasis and results in a procoagulant state that causes thrombin formation and fibrin deposition. Thrombosis and vascular leaking are implicated in hypoperfusion of multiple organs and subsequent MODS, which is the initiating event of septic shock. Moreover, endotoxins and proinflammatory cytokines can also interact with endothelial cells and result in the generation and release of NO and prostacyclin, which play vital roles in vasodilation [122–124]. Vasodilation will further aggravate hypotension and accelerate the occurrence of septic shock. Both thrombosis and hypotension also impair tissue oxygenation and aggravate organ dysfunction. In addition, mitochondrial dysfunction, caused by oxidative stress and the uncoupling mitochondria respiration, impairs cellular oxygen use and the normal operation of vital organs [125]. For instance, in a septic mouse model, there is overproduction of reactive oxygen species and reactive nitrogen species, which have been shown to impact negatively on myocardial mitochondrial function and cardiomyocyte contractility [126]. The contractility of cardiomyocytes is also impaired by other mechanisms, such as the cell-surface adhesion molecule ICAM-1 [127], small calcium-regulated molecules (S100A8 and S100A9) [128] and inappropriate mitochondrial autophagy (mitophagy) [129]. In short, septic cardiomyopathy exacerbates hypoperfusion of other organs and accelerates the progression of sepsis, ultimately resulting in septic shock.

### Diagnosis of sepsis after burn trauma

Due to the high lethality of sepsis, it is particularly important to choose an appropriate definition and criteria for early diagnosis and prediction of sepsis in burn patients. Yan and coworkers compared the sensitivity of the sepsis criteria of the ABA definition, the Mann-Salinas definition, and the new Sepsis-3 consensus definition in patients with burn and found Sepsis-3 to have a higher sensitivity (85%) than the ABA (60%) and Mann-Salinas (20%) definitions. The major advantage of Sepsis-3 is that it argues that the multiple organ dysfunction is more sepsis-specific than inflammation [15]. Despite this, it remains challenging to differentiate sepsis from systemic inflammatory response syndrome because they have similar clinical manifestations in multiple aspects, including core body temperature, respiratory rate, heart rate and hyperglycemia [101, 130]. Thus, the importance of more specific diagnostic and prognostic tools of burn sepsis cannot be overemphasized. Several utilized clinical and promising predictors associated with burn sepsis are presented in this section.

#### C-reactive protein

CRP is an evolutionarily conserved protein and is produced primarily by hepatocytes following induction by inflammatory cytokines, such as IL-6 [131]. This biomarker of inflammation in acute-phase responses has been widely adopted in clinical settings. In healthy individuals, the levels of CRP in plasma are almost undetectable, while more than 500 mg/l can be observed in patients with burn trauma [132]. Its levels may further increase in burn patients with infection or sepsis [133], thus previous studies have suggested CRP as a good predictor of sepsis in burn patients. However, recent evidence has shown that CRP has drawbacks in the specific diagnosis of sepsis in severely burned patients [134, 135]. Taken together, CRP may not be a specific biomarker of sepsis, but its levels have important reference value in conjunction with other tools, such as PCT and some cytokines.

#### Procalcitonin

PCT, as a popular biomarker in bacterial infections and sepsis, has been studied extensively and utilized clinically [136, 137]. Several studies have compared PCT to CRP in the diagnosis of sepsis, and most of the evidence suggests that PCT is superior to CRP [138–140]. PCT, the prohormone of calcitonin, is a 116-amino acid polypeptide encoded by the CALC-1 gene [141]. PCT is mainly produced by neuroendocrine cells of the thyroid and its expression is inhibited in non-endocrine tissues under normal physiological conditions [142]. Bacterial infection facilitates the transcription of CALC-1 gene in non-endocrine cells and increases PCT levels to a peak during the first 20 hours after infection [143]. The increasing serum levels of PCT in patients with burn was first reported by Assicot and colleagues, who conjectured that levels of PCT are associated with the progression of infections, sepsis and septic shock [136]. Consistent of this hypothesis, Brunkhorst *et al.* showed that levels of PCT were proportional to the severity of sepsis in critically ill patients [144]. Conversely, recent studies by Seoane *et al.* and Paratz *et al.* found no association between PCT levels and sepsis in adult burn patients [145, 146]. Therefore, like CRP, PCT is not specific in early diagnosis of sepsis in burn patients.

#### Cytokines

Major burn injuries are often accompanied by an inflammatory response that results in the activation of inflammatory pathways and the augmentation of various cytokines, including proinflammatory cytokines (TNF-α, IL-6, IL-8) and anti-inflammatory cytokines (IL-10) [147]. Recently, the potential of these cytokines in the early diagnosis of sepsis post burn injury has been investigated. Compared with the burn patients without signs of sepsis, higher levels of TNF-α were observed in burn patients with sepsis [148]. This difference also appears in serum IL-6 values between the burn patients with and without sepsis [147]. In addition, a clinical study, in which 468 children with burn injuries were divided into 2 groups based on IL-8 levels, has shown the positive correlation between the serum levels of IL-8 and sepsis in pediatric patients with elevated IL-8 [149]. Interestingly, IL-10, an anti-inflammatory cytokine, has a negative impact on the production of proinflammation cytokines, whereas the elevation of serum IL-10 levels is also correlated with the development of sepsis and even the risk of mortality in burn patients [150, 151]. Taken together, these findings indicate that cytokines hold great early diagnostic potential in sepsis and further studies will be needed to verify this.

#### Promising biomarkers

Presepsin, a glycoprotein fragment produced by monocytes and macrophages, is a soluble subtype of the cluster of differentiation 14 [152]. This glycoprotein recognizes and interacts with endotoxin complexes for the activation of systemic inflammatory signaling pathways [153]. There is mounting evidence to indicate that presepsin is a promising biomarker for diagnosing sepsis in burn patients, although it cannot be used alone to confirm or exclude the presence of sepsis in burn patients [154–157]. Mid-regional pro-atrial natriuretic peptide is another promising biomarker, and Gille *et al.*, in a prospective observational study of 42 burn patients, found that burn patients with sepsis have higher levels of this peptide and PCT [158]. Moreover, Hampson *et al.* found that neutrophil function, immature granulocyte count and plasma cell-free DNA levels showed significant potential for the early diagnosis of sepsis in burn patients [159]. Especially interesting w that micro RNA can also serve as a diagnostic biomarker. An example of this is miR-495, which is significantly downregulated in patients with sepsis and negatively correlated with CRP and PCT [160]. Although numerous promising biomarkers of sepsis have been discovered, none of them alone can diagnose sepsis post burn injury, and their values must be interpreted with caution to ensure accurate diagnosis.

## Conclusions

Sepsis and septic complications not only account for the poor outcomes in burn patients, but also prolonged hospital stays and higher medical costs. Burn wound infection is a major cause of sepsis development in patients with severe burns. Moreover, other events following burn injury play important roles in the occurrence of sepsis, such as vascular leak, hypovolemia, hypermetabolism and immune dysregulation. Integrated management, including, but not limited to, fluid resuscitation, nutritional support, antimicrobial therapy and vasoactive medications is beneficial for the prevention and prognosis of sepsis by targeting the events leading to sepsis following burn injury. However, the prediction and diagnosis of sepsis or infection remains an ongoing challenge in burn patients, although numerous predictors for burn sepsis have been reported. More investigations are needed to explore novel diagnostic tools of burn sepsis due to the unreliability and limitation of the established biomarkers (CRP, PCT and cytokines). Meanwhile, Sepsis-3, which can be applied for analysis or research purposes, appeared to be a better definition of sepsis. Based on this definition and patient-specific molecular and biochemical profiles, clinicians can design an individualized management strategy which may improve the prognosis of burn patients with sepsis.

## Abbreviations

MODS: multiple organ dysfunction syndrome; TBSA: total body surface area; Sepsis-3: Third International Consensus Definition for Sepsis and Septic Shock; CRP: C-reactive protein; PCT: procalcitonin; VEGF: vascular endothelial growth factor; ROCK: Rho-associated protein kinase; NO: nitric oxide; VE-cadherin: vascular endothelial cadherin; TNF: tumor necrosis factor; NK: natural killer; WAT: white adipose tissue; ABA: American Burn Association; ABA: American Burn Association; ATP: adenosine triphosphate; CRP: C-reactive protein; DAMPs: damage-associated molecular patterns; ER: endoplasmic reticulum; IL: interleukins; MAP: mean arterial pressure; MODS: multiple organ dysfunction syndrome; NK: natural killer; NO: nitric oxide; PAMPs: pathogen-associated molecular pattern molecules; PCT: procalcitonin; ROCK: Rho-associated protein kinase; Sepsis-3: Third International Consensus Definition for Sepsis and Septic Shock; SOFA: Sequential Organ Failure Assessment; TBSA: total body surface area; Th-1: helper T lymphocyte; Th-2: helper T lymphocyte 2; Th-17: helper T lymphocyte 17; TNF: tumor necrosis factor; TJ: tight junction; VE-cadherin: vascular endothelial cadherin; VEGF: vascular endothelial growth factor; WAT: white adipose tissue.
